# Editorial: The Neurovascular Unit as a Potential Biomarker and Therapeutic Target in Cerebrovascular Disease

**DOI:** 10.3389/fnagi.2022.908716

**Published:** 2022-05-05

**Authors:** Shereen Nizari, Cheryl A. Hawkes, Yorito Hattori, Anusha Mishra

**Affiliations:** ^1^Centre for Advanced Biomedical Imaging, Division of Medicine, University College London, London, United Kingdom; ^2^Centre for Cardiovascular and Metabolic Neuroscience, Department of Neuroscience, Physiology & Pharmacology, University College London, London, United Kingdom; ^3^Department of Biomedical and Life Sciences, Lancaster University, Lancaster, United Kingdom; ^4^Department of Neurology, National Cerebral and Cardiovascular Center, Suita, Japan; ^5^Knight Cardiovascular Institute, Oregon Health & Science University, Portland, OR, United States; ^6^Department of Neurology, Jungers Center for Neurosciences Research, Oregon Health & Science University, Portland, OR, United States

**Keywords:** neurovascular unit (NVU), cerebrovascular disease, biomarker, cerebral blood flow (CBF), therapeutic target

## Introduction

The brain is one of the most metabolically demanding organs—it requires 20% of the body's energy supply yet forms only 2% of the body's weight. Therefore, brain activity is supported by a dynamically regulated supply of blood and, hence, energy substrates through the cerebrovascular network. Furthermore, the post-mitotic nature of neurons and their longevity makes the brain very sensitive to extracellular perturbations. Thus, the cerebrovasculature also has to be specialized to minimize perturbations of the brain microenvironment, a property mediated by the blood-brain barrier that is formed by the neurovascular unit (NVU).

The NVU is a multicellular structure formed by neurons, glia and vascular cells. The NVU is fundamental to the distribution of cerebral blood flow, regulation of extracellular homeostasis and, as recently shown, waste clearance from the brain. Therefore, the NVU represents a pivotal point of vulnerability for the brain. In this Research Topic, 12 contributing articles address the potential of the NVU and provide evidence demonstrating the importance of the NVU as a biomarker and therapeutic target for cerebrovascular disease.

## Physiological Parameters of the NVU as Biomarkers and Targets for Therapeutic Intervention

It is important to first understand the structure and function of the healthy NVU to identify abnormal events and therapeutic targets. Shaw et al. provide a thorough and detailed characterization of the cerebrovascular bed, highlighting differences in its structure and function as it branches from penetrating arterioles to capillaries. Using immunohistochemistry and by assessing vasomotion along the vascular tree in awake mice *via* two-photon microscopy, they provide novel data characterizing points of contractility, angiogenic capacity, and vasodilatory potential across the vascular tree and branch points (Shaw et al.). The differences in vasodilatory response across the vascular tree are also highlighted in the study by Rosehart et al., in which they show that stimulation of capillaries with prostaglandin E2 elicits vasodilation of upstream arterioles. This was blunted in a model of small vessel disease, suggesting a reduction in capillary-to-arteriole signal propagation as a potential mechanism for the pathologies associated with cerebral small vessel disease, while also suggesting a new biomarker for NVU dysfunction (Rosehart et al.).

Specific modifiable physiological parameters of the NVU are also highlighted within this Research Topic. Inhibitors of carbonic anhydrases, the pH regulating enzymes, can protect the NVU in stroke and Alzheimer's disease, as reviewed by Lemon et al. Hayes et al. provide novel data on another parameter, the neuroprotective hormone insulin-like growth factor-1 (IGF-1), demonstrating a difference in vulnerability to glutamate-induced toxicity across different cell types of the NVU when IGF-1 is inhibited.

Together, these data highlight multiple structural and functional points within the NVU that may potentially serve as targets for therapeutic intervention. The differential regulation at the various vascular segments and the transitional zones between them in the vascular tree is also noteworthy; each segment could be differently affected during disease, or be targeted for therapy.

## NVU Risk Factors and BIOMARKERS Across the Lifespan

The biomarker and target potential of the NVU across the lifespan is another key focus of this topic. In an opinion piece, Beishon and Panerai emphasize the importance of mid-life therapeutic targeting and lifestyle alterations targeting known risk factors for dementia. The comprehensive review by Ouellette and Lacoste highlights the shared vascular abnormalities associated with neurodevelopmental and neurodegenerative disorders, despite their distinct clinical presentation at different life stages.

Lecordier et al. provide a detailed review of early risk factor events for NVU dysfunction leading to dementia, highlighting triggering pathological events. They specifically underscore the importance of air pollution as a risk factor for dementia, and provide a detailed summary of its impact on NVU components (Lecordier et al.). Exercise is suggested to reduce cerebrovascular disease and alleviate the risk for developing dementia. Ohtomo et al. report that, in a model of chronic hypoperfusion, exercise was able to alleviate fewer behavioral deficits in middle aged mice compared to their previous study on young mice, suggesting the value of early lifestyle interventions. Altogether, these reports showcase the potential of the NVU as a biomarker and the importance of protecting NVU function across the lifespan.

## Clinical Biomarkers of Disease Outcome and Progression

The significance of using the NVU, or the pathologies associated with it, as a biomarker and therapeutic target in the clinical setting is also emphasized in this Research Topic. A systematic meta-analysis by Wang et al. demonstrates the role of cerebral small vessel disease in negative prognosis following intravenous thrombolysis treatment for acute ischemic stroke, highlighting the value of NVU pathology in predicting treatment outcomes.

In addition, Monteiro et al. report a reduction in neurovascular coupling in patients with hypertension while hypercapnic hyperemia is preserved, prior to detectable cerebral small vessel disease. This impairment was further exacerbated when hypertension was comorbid with diabetes, suggesting that NVU dysfunction may contribute to the cerebral effects of chronic hypertension and diabetes (Monteiro et al.). Staszewski et al. provide further support for the NVU as a biomarker for disease progression, demonstrating that decreases in cerebrovascular reactivity are measurable over a 24 month follow-up period in patients with small vessel disease, irrespective of initial radiological disease assessment.

Lastly, Ren et al. suggest that disruptions in the glymphatic system, a hypothesized route of paravascular waste clearance, may be the cause of, and serve as a biomarker for, peri-operative neurocognitive disorder. Their review highlight anesthesia choice and management as potentially modifiable risk factors for peri-operative neurocognitive disorder (Ren et al.).

## Conclusion and Future Directions

This Research Topic provides both preclinical and clinical evidence of the importance of the NVU as a contributor to cerebrovascular disease and, thus, a therapeutically targetable fulcrum, while underscoring its value as an accessible biomarker for disease etiology and progression. The additional targeting of co-morbidities and detrimental lifestyle risk factors that lead to NVU dysfunction, which change over the lifespan, are also essential for disease management. It is clear that further characterization of the NVU, including its functions in health and different pathological contexts, and how it is modified by lifestyle choices and comorbid conditions, are all important avenues for future work. Findings from such studies may suggest potential therapeutic targets for cerebrovascular diseases, and may even be applicable to related dementia conditions.

## Author Contributions

All authors listed have made a substantial, direct, and intellectual contribution to the work and approved it for publication.

## Conflict of Interest

The authors declare that the research was conducted in the absence of any commercial or financial relationships that could be construed as a potential conflict of interest.

## Publisher's Note

All claims expressed in this article are solely those of the authors and do not necessarily represent those of their affiliated organizations, or those of the publisher, the editors and the reviewers. Any product that may be evaluated in this article, or claim that may be made by its manufacturer, is not guaranteed or endorsed by the publisher.

